# Improved Lanthipeptide Detection and Prediction for antiSMASH

**DOI:** 10.1371/journal.pone.0089420

**Published:** 2014-02-20

**Authors:** Kai Blin, Daniyal Kazempour, Wolfgang Wohlleben, Tilmann Weber

**Affiliations:** 1 Microbiology/Biotechnology, Interfaculty Institute of Microbiology and Infection Medicine, Eberhard-Karls-University of Tübingen, Tübingen, Germany; 2 German Centre for Infection Research, Tübingen, Germany; Russian Academy of Sciences, Institute for Biological Instrumentation, Russian Federation

## Abstract

Lanthipeptides are a class of ribosomally synthesised and post-translationally modified peptide (RiPP) natural products from the bacterial secondary metabolism. Their name is derived from the characteristic lanthionine or methyl-lanthionine residues contained in the processed peptide. Lanthipeptides that possess an antibacterial activity are called lantibiotics. Whereas multiple tools exist to identify lanthipeptide gene clusters from genomic data, no programs are available to predict the post-translational modifications of lanthipeptides, such as the proteolytic cleavage of the leader peptide part or tailoring modifications based on the analysis of the gene cluster sequence. antiSMASH is a software pipeline for the identification of secondary metabolite biosynthetic clusters from genomic input and the prediction of products produced by the identified clusters. Here we present a novel antiSMASH module using a rule-based approach to combine signature motifs for biosynthetic enzymes and lanthipeptide-specific cleavage site motifs to identify lanthipeptide clusters in genomic data, assign the specific lanthipeptide class, predict prepeptide cleavage, tailoring reactions, and the processed molecular weight of the mature peptide products.

## Introduction

### Lanthipeptides

Lanthipeptides are polycyclic peptides named after the thioether-linked amino acids lanthionine and (2 *S*,3 *S*,6 *R*)-3-methyllanthionine contained in the mature peptide. Formerly called lantibiotics from “lanthionine-containing antibiotics”, the new name lanthipeptide was proposed to also include non-antibiotic peptides of the same biosynthetic origin [1]. The best-known lanthipeptide is Nisin, which has been used as a food preservative since the 1940 s. Still a hot spot of natural products research, other lanthipeptides, for example NVB302, entered phase 1 clinical trials in 2011 as a treatment against *Clostridium difficile* infections [2]. Lanthipeptides are ribosomally synthesised and post-translationally modified peptides (RiPPs). The extensive post-translational modifications enhance the stability of the mature peptide against proteolysis and temperature stress. Lanthipeptides are encoded on the genome as a precursor peptide containing a leader and a core peptide part. The leader peptide, which is removed at a very late stage of the biosynthesis by a cluster-encoded or host-encoded protease, serves as a docking site for the modifying enzymes acting on the core peptide [3]. The lanthionine (Lan) and methyllanthionine (MeLan) residues are introduced in a two-step reaction. First, serine (Ser) and threonine (Thr) residues are dehydrated to dehydroalanine (Dha) and dehydrobutyrine (Dhb), respectively, usually with an intermediate phosphorylation step. In the second step, a Michael-type addition by cysteine (Cys) residues onto the dehydro amino acids then yields the thioether cross-links.

Depending on the biosynthetic enzymes installing the thioether cross-links, lanthipeptides are divided into different classes [4]. Currently, four lanthipeptide classes are known. In class I lanthipeptides, the dehydration is catalysed by a dedicated dehydratase commonly called LanB. Cyclisation is carried out by a cyclase called LanC. In specific gene clusters, the generic enzyme names might be replaced by a more specific name: for example in the nisin gene cluster, the LanB-type dehydratase is called NisB and the LanC-type cyclase is called NisC. For the remaining class II, III and IV lanthipeptides, both dehydration and cyclisation are catalysed by a single bi-functional enzyme. A class II LanM enzyme carries an N-terminal dehydratase domain with little sequence similarity to other characterised enzymes. The C-terminal cyclisation domain is similar to the LanC enzymes from class I lanthipeptide cyclases. The bi-functional enzymes for class III (LanKC) and IV (LanL) have a common N-terminal phospho- serine/phosphothreonine lyase domain and a central kinase domain. The C-terminal cyclisation domain in class III enzymes, while similar to the cyclisation domains from the other classes, lacks three zinc- bindinding residues that are conserved in the other classes. In class IV, those residues are present. In addition to the introduction of Lan and MeLan, a number of further post-translational modifications may occur if the appropriate tailoring enzymes are present in the gene cluster. Among the modifications found in lanthipeptides is the formation of *S*-[(*Z*)-2-aminovinyl]-D-cysteine (AviCys) or *S*-[(*Z*)-2-aminovinyl]-(3 *S*)-3-methyl-D-cysteine (AviMeCys) [5]. The formation of AviCys and AviMeCys is catalysed by an enzyme of the family of homo-oligomeric flavin-containing cysteine decarboxylases.The enzyme with the generic designation LanD catalyses the oxidative decarboxylation of a C-terminal cysteine residue to a reactive thio–enol intermediate, which then cyclises with a Dha or Dhb residue, respectively, yielding AviCys or AviMeCys. An example would be the AviCys residue in epidermin introduced by EpiD [6]. Another post-translational modification is the chlorination of tryptophan residues catalysed by a flavin-dependent tryptophan halogenase designated LanH. This kind of reaction has been observed in the chlorination of tryptophan by MibH in microbisporicin biosynthesis [7]. If the cluster contains a cytochrome P450 oxygenase designated LanO, amino acids in the modified precursor peptide can be hydroxylated, as observed in the hydroxylation of proline in microbisporicin biosynthesis [7]. If the N-terminal amino acid is Dha and an oxidoreductase is present in the cluster, the N-terminal amino acid can be converted to lactate, as observed in the epicidin 280 cluster [8].

### antiSMASH

antiSMASH, the antibiotics and secondary metabolite analysis shell, is a software pipeline for the automated identification of secondary metabolite biosynthesis clusters in whole genome sequenes of bacteria and fungi [9], [10]. In antiSMASH, the user, who does not need specialized bioinformatics training, can upload microbial genome sequences which then are mined for secondary metabolite biosynthetic pathways in a fully automated manner. In the initial release of antiSMASH, the prediction of the products of the biosynthetic pathways was only possible for non-ribosomal peptide synthase (NRPS) and polyketide synthase (PKS) gene clusters [9]. In 2013, we released antibiotics and secondary metabolite analysis shell (antiSMASH) 2.0 [10]. In the new release, the architecture of the software was redesigned, now making it possible to add new predictors as self-contained plug-ins. antiSMASH is available as a web service at http://antismash.secondarymetabolites.org and can also be downloaded to run standalone. It is released under the GNU Affero Public License version 3, an OSI-approved Open Source license. Here we present the implementation of a lanthipeptide-specific analysis module for antiSMASH 2. With this module, lanthipeptide biosynthetic pathways can be automatically detected, classified and their products predicted. In addition, the module can identify putative protease cleavage sites for the most abundant class I and class II lanthipeptides and predict the molecular masses of the final products. The module is shipped with the antiSMASH 2.2 release and is also running on the public web server.

## Design, Implementation and Validation

Secondary metabolite clusters in antiSMASH are identified using Hidden Markov Models (HMMs) of protein motifs for key biosynthetic enzymes. Which profiles are required to be identified for a specific secondary metabolite type is described by a rules file containing one rule-set per cluster type. Rule-sets can be simple hits against a single profile, AND and OR combinations of multiple profiles, or a selection of more complex rules, e.g. requiring a match against a minimum of n hits of a set of profiles. New secondary metabolite types can be included by adding new profile HMMs and extending the rules file. Once the cluster detection has identified a secondary metabolite cluster of a certain type, specific analysis modules can be run to generate a more detailed analysis of the pathway and the prediction of the product of a given cluster. Specific analysis modules are written as self-contained plug-ins that are loaded from the user’s PYTHONPATH at run-time.

### Identification of Lanthipeptide Biosynthetic Gene Clusters

To make more detailed cluster information available to the downstream specific analysis module, the cluster detection rules have been extended to include domain-specific Pfam [11] HMMs for the N-terminal domain (PFAM: PF13575) of class II LanM enzymes, the central kinase domain (PFAM: PF00069) of class III and IV enzymes, LanD-type flavin-dependent decarboxylases (PFAM: PF02441), LanH-type flavin-dependent halogenases (PFAM: PF04820), LanO-type cytochrome P450 oxygenases (PFAM: PF00067) and EciO-type short chain dehydrogenases (PFAM: PF00106, PF13561) (see Table 1 for details). Cutoff-values were determined empirically by analysing known sequences with the HMM profiles and manually adjusted to gain optimal sensitivity while keeping false positive hits low.

**Table 1:** Lanthipeptide-related HMM profiles and scores.

Name	Description	Cutoff	File
LANC_like	LanC-like lantibiotics biosynthesis protein	17	LANC_like.hmm
DUF4135	Lantibiotic-associated domain	150	PF13575.hmm
Lant_dehyd_N	Lantibiotic dehydratase, N-terminus	20	Lant_dehyd_N.hmm
Lant_dehyd_C	Lantibiotic dehydratase, C-terminus	20	Lant_dehyd_C.hmm
Flavoprotein	Lantibiotic aminovinyl flavoprotein	20	PF0241.hmm
Trp_halogenase	Tryptophan halogenase	20	PF04820.hmm
p450	P450 oxygenase	60	PF00067.hmm
Pkinase	Protein kinase domain	30	PF00069.hmm
adh_short	Short-chain dehydrogenase	100	PF00106.hmm
adh_short_C2	Short-chain dehydrogenase, C-terminus	100	PF13561.hmm
Antimicr18	Lantibiotic antimicrobial peptide 18	20	Antimicrobial18.hmm
Gallidermin	Gallidermin	20	Gallidermin.hmm
L_biotic_A	Lantibiotic, type A	20	L_biotic_typeA.hmm
TIGR03731	Lantibiotic, gallidermin/nisin family	18	TIGR03731.hmm
leader_d	Lantibiotic leader lacticin 481 group	20	LE-LAC481.hmm
leader_eh	Lantibiotic leader mersacidin cinnamycin group	20	LE-MER+2PEP.hmm
leader_abc	Lantibiotic leader LanBC modified	20	LE-LanBC.hmm
mature_d	Lantibiotic peptide lacticin 481 group	20	MA-LAC481.hmm
mature_ab	Lantibiotic peptide nisin epidermin group	20	MA-NIS+EPI.hmm
mature_a	Lantibiotic peptide nisin group	20	MA-NIS.hmm
mature_b	Lantibiotic peptide epidermin group	20	MA-EPI.hmm
mature_ha	Lantibiotic peptide two component alpha	20	MA-2PEPA.hmm
mature_h_beta	Lantibiotic peptide two component beta	20	MA-2PEPB.hmm
lacticin_l	lantibiotic leader lacticin 481 group (Dufour et al)	20	LE-DUF.hmm
lacticin_mat	lantibiotic peptide lacticin 481 group (Dufour et al)	20	MA-DUF.hmm
LD_lanti_pre	FxLD family lanthipeptide	20	TIGR04363.hmm
strep_PEQAXS	*Streptomyces* PEQAXS motif lanthipeptide	20	strep_PEQAXS.hmm

### Prediction of the Lanthipeptide Class

Lanthipeptide classes are assigned by determining the domains present in the biosynthetic enzymes. Characteristic for class I lanthipeptides is the separate LanB enzyme containing the dehydratase domain, so the class prediction checks for a hit against the Lant_dehyd_N (PFAM: PF04737) or Lant_dehyd_C (PFAM: PF04738) domains. The dehydratase domain of class II LanM-type enzymes is characteristic as well, so if the cluster contains this dehydratase domain (PFAM: PF13575), the lanthipeptide will be considered class II. Class III LanKC-type and class IV LanL-type enzymes are identified via the central kinase domain (PFAM: PF00069). To differentiate between class III and IV enzymes, the algorithm checks if the conserved zinc binding sites in the C-terminal cyclase domain are absent (class III) or present (class IV).

### Cleavage Site prediction

In the final step in lanthipeptide biosynthesis, a protease cleaves the leader peptide part off the modified precursor peptide to yield the mature peptide. Depending on the class of the lanthipeptide, the cleavage site motives vary widely. In order to predict the cleavage site, we have created a manually curated set of HMMs, one for lanthipeptide classes I and II each (Tables 2, 3). Profiles for the HMMer 2.3.2 software [12] were generated using the command hmmbuild profile.hmm alignment.fa; hmmcalibrate profile.hmm. As the method depends on the size of the seed sequence data set, we decided not to include cleavage site predictions for class III (only six seed sequences available) and class IV (no experimentally verified sequences available) lanthipeptides. Once more seed sequences become available for these two classes, adding cleavage site predictions using the same method will be straightforward.

**Table 2:** Class I cleavage site motif sequences.

Name	Position	Sequence
**mutacin_1140**	22…41	FAFDTTDTTIVASNDDPDTR
**mutacin_Ny266**	22…41	FTFDTTDTIVAESNDDPDTR
**salivaricin_D**	6…23	FNLDLVEVSK–SNTGASAR
**nisin_U**	6…24	FNLDLIKISK-ENNSGASPR
**nisin_A**	6…23	FNLDLVSVSKK–DSGASPR
**nisin_Z**	6…23	FNLDLLSVSKK–DSGASPR
**nisin_Q**	6…23	FNLDLVSVSKT–DSGASTR
**gallidermin**	11…30	FDLDVKVNAKESNDSGAEPR
**epidermin**	11…30	FNLDVKVNAKESNDSGAEPR
**entianin**	7…24	FDLDVVKVSKQ–DSKITPQ
**Pep5**	8…26	FDLEIKKETSQNTD-ELEPQ
**epicidin_280**	8…26	FDLEIKKDNME-NNNELEPQ
**epilancin_K7**	6…24	FDLNLNKGVETQK-SDLSPQ
**geobacillin_I**	7…23	FDLDIVVK-KQ–DDVVQPN
**streptin**	8…23	FDLDLKTNKK–D-TATPY
**microbisporicin**	17…33	LDLDLSIGVEE–ITAGPA

**Table 3:** Class II cleavage site motif sequences.

Name	Position	Sequence
**mutacin_II**	19…52	EL-TILGG
**variacin**	15…47	ELDAILGG
**salivaricin_A**	22…51	ELMEVAGG
**butyrivibriocin**	16…48	ELEQILGG
**streptococcin_A_FF22**	20…51	ELDNLLGG
**lichenicidin_A1**	30…74	EQHSIAGG
**lichenicidin_A2**	27…72	ELKALVGG
**thermophilin_1277**	18…66	ELEMLIGG
**lacticin_A1**	16…59	FDEDVFGA
**lacticin_A2**	26…65	EGDESHGG
**nukacin_KQ_131**	23…57	ELNEVLGA
**macedocin**	18…51	ELDQIIGA
**salivaricin_B**	24…56	ELDNVLGA
**haloduracin_A1**	33…69	ILAGVNGA
**haloduracin_A2**	28…65	ELSSLAGS
**cytolysin**	12…68	EMEAIQGS
**plantaricin_W**	24…59	NLLNVNGA
**cinnamycin**	52…59	IAATEAFA
**mersacidin**	41…48	QMDKLVGA
**actagardine**	35…64	EDRTIYAA
**michiganin_A**	36…66	RRVVSPYM

### Monoisotopic Mass, Molecular Weight and Alternative Weights

Once the cleavage site is predicted, both the monoisotopic mass and the average molecular weight are calculated. For the calculation of these numbers it is assumed that all Ser and Thr residues are dehydrated to Dha and Dhb respectively. As a lack of dehydration is frequently observed but the mechanism behind this has not been elucidated, we also calculate alternative weights under the assumption that one up to *n* Ser or Thr are not dehydrated, where *n* is the number of Ser and Thr residues in the core peptide subtracted by the number of Cys residues in the core peptide. This upper bound is set to account for the observation that all Cys residues tend to participate in Lan or MeLan bridges with Dha or Dhb residues.

### Predicting Tailoring Reactions

Tailoring reactions are not performed by the core biosynthetic enzymes that perform the dehydration and cyclisation but instead by additional enzymes also encoded on the cluster.

#### AviCys and AviMeCys formation

The unusual amino acids AviCys and AviMeCys are formed by oxidative decarboxylation of the C-terminal Cys residue. The resulting thio–enol intermediate cyclises with a Dha or Dhb side-chain respectively. This reaction is catalysed by a LanD-type flavin-dependent decarboxylase, identified by a hit against the PFAM PF02441 profile with a score ≥20. The formation of AviCys or AviMeCys reduces the predicted peptide weight by 46 Da.

#### Halogenation

LanH-type halogenases chlorinate an amino acid side chain, increasing the predicted peptide weight by 34 Da. These enzymes are identified by a hit against the PFAM PF04820 profile with a score ≥20.

#### Hydroxylation

LanO-type cytochrome P450 oxygenases catalyse the regiospecific oxidation of non-activated hydrocarbons. The enzymes are identified by a hit against PFAM PF00067 with a score ≥60. The hydroxylation increases the predicted peptide weight by 16 Da.

#### Lactate formation

EciO-type short-chain dehydrogenases, identified by a hit against PFAM PF00106 or PFAM PF13561 with a score ≥100, catalyse the final step of the conversion of the N-terminal Dha residue to lactate. This increases the predicted peptide weight by 2 Da.

#### Predicting the number of Lan and MeLan bridges

To predict the number of Lan and MeLan bridges, a simple heuristic is applied using the formula.
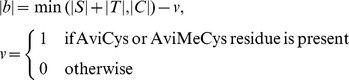
where |b| is the number of bridges, and |S|, |T|, |C| is the number of amino acids Ser, Thr, and Cys in the core peptide.

### Validation and Benchmarking

To validate the robustness of the cleavage site HMM-profiles, we used *n-fold cross validation*. For a seed alignment of size *n*, we built *n* different profiles by including *n − 1* sequences, and then checked if a cleavage site was predictable and correct for the left out sequence. A cleavage site was predictable if the profile produced a hit with a score above the threshold. A cleavage site was considered correct if the prediction matched the ungapped seed sequence not used for building the profile. A cleavage site was predicted in all test sequences for both class I and II lanthipeptides. Cleavage sites were correct in all class I test inputs and in 81% of the class II test inputs (Table 4). To benchmark the overall performance of the prediction, we ran a number of lanthipeptide biosynthetic gene clusters through antiSMASH. We checked if the gene cluster was identified, the precursor peptide was detected, and finally the peptide mass was predicted correctly.

**Table 4:** Stability of the prediction motifs.

Class	# of Sequences	# Found	% Found	# Correct	% Correct
**I**	16	16	1.00	16	1.00
**II**	21	21	1.00	17	0.81

Among the clusters run for benchmarking, we included the planosporicin and epilancin 15X clusters. The cleavage sites of both of these lanthipeptides were not part of the seed alignments. For planosporicin, the algorithm predicted a mass of 2193.3 Da (actual mass 2194 Da [13]) and correctly predicted 5 lanthionine bridges. For epilancin 15X, the algorithm predicted a mass of 3171.8 Da (actual mass 3171.7 Da [14]) and correctly predicted 3 lanthionine bridges and the N-terminal lactate.

## Results and Discussion

Only few tools are currently available that allow the automated identification of RiPPs. Apart from antiSMASH, there is BAGEL, recently released in version 3 [15]. BAGEL targets a large number of different ribosomally synthesised peptides. For lanthipeptides, BAGEL only predicts the leader peptide and the class, but does not attempt to predict tailoring reactions, number of Lan and MeLan bridges or the molecular weight.

The detection of enzymes responsible for tailoring reactions is central in the prediction of the mature peptide mass. antiSMASH can predict the presence of AviCys residues which are found, for example, in epidermin and microbisporicin. Halogenations and hydroxylations, which occur in microbisporicin biosynthesis are also detected. A remaining issue in mass prediction is that not all Ser and Thr residues are dehydrated in all the peptides, resulting in mass predictions that are 18 Da too low per undehydrated amino acid. antiSMASH assists in detecting the presence of undehydrated residues by providing alternative mass predictions for lanthipeptides that carry more Ser and Thr than Cys residues. Once the tailoring reactions have been predicted, the final step is the prediction of the number of Lan and MeLan bridges. The naïve heuristic of counting the Cys and Ser/Thr residues and then using the smaller number fails if the mature peptide contains an AviCys or AviMeCys residue and needs to be adjusted accordingly.

**Figure 1:**
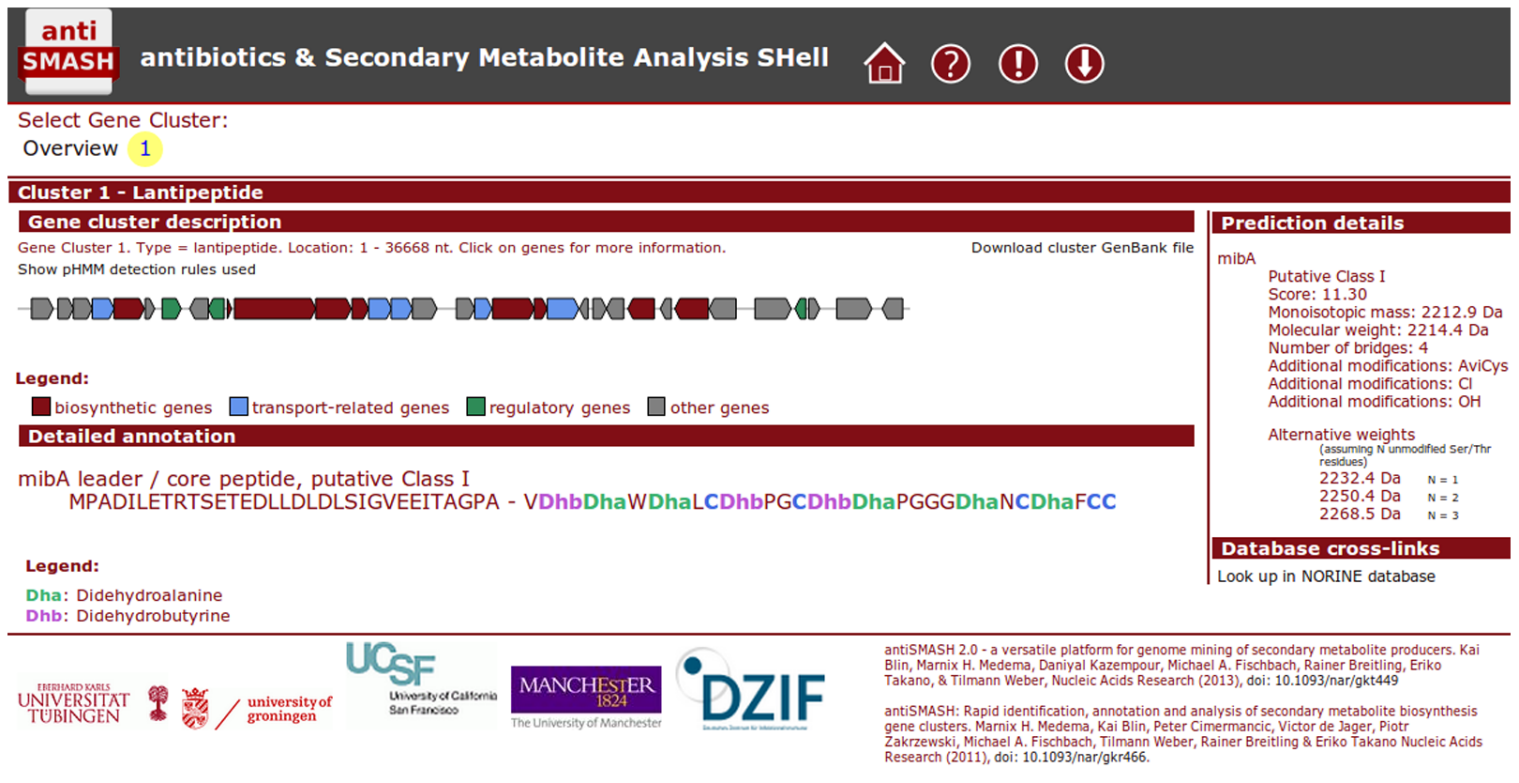
Example lanthipeptide output antiSMASH 2.2 output for the microbisporicin [5] gene cluster, showing the predicted leader/core peptide split and the predicted tailoring reactions and weights in the sidebar.

The prediction details of the lanthipeptide module are included in the standard antiSMASH output. For the HTML output (Figure 1), lanthipeptide class and leader/core peptide split predicted are shown in the “detailed annotation” section of the cluster page. The score of the class prediction, predicted monoisotopic mass and molecular weights, the number of bridges and the identified additional modifications are shown in the “prediction details” sidebar.

To test the accuracy of the antiSMASH lanthipeptide predictions, a benchmark dataset was analyzed and the results evaluated:

The determination of the lanthipeptide class based on the biosynthetic enzymes in the cluster is straightforward, and antiSMASH performed this tasks flawlessly on the benchmark data set (Table 5). Using the advanced heuristic, antiSMASH was able to correctly predict the number or bridges in almost all lanthipeptides of the benchmark data-set. The heuristic only fails if two Cys residues form a disulphide bridge, a rare occurrence observed in e.g. thermophilin 1277 [16]. Unfortunately, the enzyme catalysing the formation of the disulphide bridge is not present in the gene cluster and thus cannot be used to predict a disulphide bridge formation [16]. Predicting the core peptide sequence is more difficult. The cleavage site motif of class II lanthipeptides (Table 3) is relatively uniform, largely consisting in two amino acids with small side chains that are preceded by alternating hydrophobic and hydrophilic residues. In fact, the four class II cleavage sites incorrectly predicted during validation (Table 4) all differed from this pattern and contained a site that more closely matches the motif upstream of the actual cleavage site. Some class II core peptides like mersacidin [17] or lichenicidin A2 [18] lose an additional six amino acids at the N-terminus after the proteolytic cleavage [18], so it seems likely that the predicted cleavage sites may be accurate and some additional enzyme catalyses the N-terminal modifications. Class I leader peptides also carry a short motif of alternating hydrophobic and hydrophilic amino acids, usually called the FNLD motif. The spacer between this motif and the actual cleavage site varies. At position −2 in front of the cleavage site, many leader peptides carry a proline residue (Table 2). During validation, all cleavage sites were predicted correctly (Table 4). Due to the strong signal of the FNLD motif, class I prediction (stability 100%) is even more robust than the class II prediction (stability 81%) with the shorter motif. As a proof of concept, we used the gene clusters of the recently published planosporicin [13] and the lactate-containing epilancin 15X [14]. Both precursor peptides contain cleavage sites that are distinct from all the sequences included in the class I seed alignment. For both lanthipeptides the algorithm is able to correctly predict the mass, number of Lan/MeLan bridges and tailoring modifications.

**Table 5:** Benchmark results.

Substance	Class	Predicted Mass (Da)	Actual Mass (Da)	Number of bridges	Source
**Salivaricin D**	I	3466.7	3467.5	4	[19]
**Nisin U**	I	3029.6	3029.0	5	[20]
**Nisin A**	I	3353.9	3354.5	5	[21]
**Nisin Z**	I	3330.9	3331.5	5	[21]
**Nisin Q**	I	3326.9	3327.3	5	[21]
**Gallidermin**	I	2164.0	2164.0	4	[6]
**Epidermin**	I	2164.0	2164.0	4	[6]
**Entianin**	I	3346.7	3346.0	5	[22]
**Pep5**	I	3487.1	3488.0	3	[6]
**Epicidin 280**	I	3135.6	3135.0	3	[8]
**Geobacillin I**	I	3261.5	3265.0	7	[23]
**Streptin 1'**	I	2441.9	2442.0	3	[24]
**Microbisporicin A2**	I	2232.4	2232.0	5	[25]
**Mutacin II**	II	3243.5	3244.0	3	[26]
**Salivaricin A2**	II	2366.6	2368.0	3	[27]
**Streptococcin A-FF22**	II	2796.1	2795.0	3	[28]
**Lichencidin A1**	II	3250.7	3251.0	4	[18]
**Lichencidin A2**	II	3632.8†	3021.0	4	[18]
**Thermophilin 1277**	II	3395.9‡	3428.0	2‡	[16]
**Lacticin 3147 A1**	II	3322.6	3322.3	4	[29]
**Lacticin 3147 A2**	II	2843.2	2847.5	3	[29]
**Salivaricin B**	II	2733.1	2740.0	3	[30]
**Cinnamycin**	II	2043.2	2041.0	3	[31]
**Mersacidin**	II	2399.0†	1826.3	3	[32]
**Actagardine**	II	1856.2	1860.5	4	[33]
**Michiganin A**	II	2145.5	2145.0	4*	[34]
**Planosporicin**	I	2193.3	2194.0	5	[13]
**Epilancin 15X**	I	3171.8	3171.7	3	[14]

† N-terminal removal of six amino acids not predicted.

‡ Contains a disulphite bridge.

* Not shown experimentally.

## Conclusions

With the algorithm described in this paper, antiSMASH gains extensive lanthipeptide-specific predictive capabilities. antiSMASH is the only software currently available that will predict lanthipeptide class, core peptide cleavage, tailoring reactions, number of Lan and MeLan bridges, and the molecular weight of the mature peptide product. These informations obtained by analyzing the genome sequence of the producers can give crucial hints for the identification of the lanthipeptide compounds in chemical analytics.
