# Correction: Improved Lanthipeptide Detection and Prediction for antiSMASH

**DOI:** 10.1371/journal.pone.0103665

**Published:** 2014-07-25

**Authors:** The PLOS ONE Staff

A column is missing from Table 1 and two of the references contained in the corrected Table 1 do not appear in the published article. Please see the complete, corrected Table 1 here.

**Table 1:** Lanthipeptide-related HMM profiles and scores.

Name	Description	Cutoff	File	Reference
LANC_like	LanC-like lantibiotics biosynthesis protein	17	LANC_like.hmm	11
DUF4136	Lantibiotic-associated domain	150	PF13675.hmm	11
Lant_dehyd_N	Lantibiotic dehydratase, N-terminus	20	Lant_dehyd_N.hmm	11
Lant_dehyd_C	Lantibiotic dehydratase, C-terminus	20	Lant_dehyd_C.hmm	11
Flavoprotein	Lantibiotic aminovinyl flavoprotein	20	PF0241.hmm	11
Trp_halogenase	Tryptophan halogenase	20	PF04820.hmm	11
p450	P450 oxygenase	60	PF00067.hmm	11
Pkinase	Protein kinase domain	30	PF00069.hmm	11
adh_short	Short-chain dehydrogenase	100	PF00106.hmm	11
adh_short_C2	Short-chain dehydrogenase, C-terminus	100	PF13661.hmm	11
Antimicr18	Lantibiotic antimicrobial peptide 18	20	Antimicrobial18.hmm	11
Gallidermin	Gallidermin	20	Gallidermin.hmm	11
L_biotic_A	Lantibiotic, type A	20	L_biotic_typeA.hmm	11
TIGR03731	Lantibiotic, gallidermin/nisin family	18	TIGR03731.hmm	35
leader_d	Lantibiotic leader lacticin 481 group	20	LE-LAC481.hmm	36
leader_eh	Lantibiotic leader mersacidin cinnamycin group	20	LE-MER+2PEP.hmm	36
leader_abc	Lantibiotic leader LanBC modified	20	LE-LanBC.hmm	36
mature_d	Lantibiotic peptide lacticin 481 group	20	MA-LAC481.hmm	36
mature_ab	Lantibiotic peptide nisin epidermin group	20	MA-NIS+EPI.hmm	36
mature_a	Lantibiotic peptide nisin group	20	MA-NIS.hmm	36
mature_b	Lantibiotic peptide epidermin group	20	MA-EPI.hmm	36
mature_ha	Lantibiotic peptide two component alpha	20	MA-2PEPA.hmm	36
mature_h_beta	Lantibiotic peptide two component beta	20	MA-2PEPB.hmm	36
lacticin_l	lantibiotic leader lacticin 481 group (according to Dufour et al., FEMS Microbiol Rev. 31(2): 134-67)	20	LE-DUF.hmm	36
lacticin_mat	lantibiotic peptide lacticin 481 group (according to Dufour et al., FEMS Microbiol Rev. 31(2): 134-67)	20	MA-DUF.hmm	36
LD_lanti_pre	FxLD family lanthipeptide	20	TIGR04363.hmm	35
strep_PEQAXS	*Streptomyces* PEQAXS motif lanthipeptide	20	strep_PEQAXS.hmm	10

Please see the additional references here.

35. Haft DH, Selengut JD, Richter RA, Harkins D, Basu MK, et al. (2013) TIGRFAMs and Genome Properties in 2013. Nucleic Acids Res 41: D387-395.

36. de Jong A, van Heel AJ, Kok J, Kuipers OP (2010) BAGEL2: mining for bacteriocins in genomic data. Nucleic Acids Res 38: W647-651.
